# Maternal, pregnancy and neonatal outcomes in triplet pregnancies in Sweden – a nationwide cohort study

**DOI:** 10.48101/ujms.v128.9473

**Published:** 2023-07-17

**Authors:** Mia-Maria Ekström, Eleonor Tiblad, Mikael Norman, Olof Stephansson, Michaela Granfors

**Affiliations:** a Clinical Epidemiology Division, Department of Medicine Solna, Karolinska Institutet, Stockholm, Sweden; b Division of Obstetrics, Department of Women’s Health, Karolinska University Hospital, Stockholm, Sweden; c Department of Clinical Science, Intervention and Technology, Karolinska Institutet, Stockholm, Sweden

**Keywords:** Triplet pregnancy, multifetal gestation, maternal outcome, perinatal outcome, fetal reduction

## Abstract

**Background:**

Triplet pregnancies carry a high risk of pregnancy-related complications. The primary aim of this study was to describe maternal, pregnancy, and neonatal outcomes in expectantly managed triplet pregnancies in Sweden. The secondary aim was to compare outcomes in expectantly managed triplet pregnancies with triplet pregnancies where fetal reduction had been performed with the only indication to reduce the number of fetuses.

**Methods:**

Nationwide cohort study based on linkage of data from three national Swedish registers. Triplet pregnancies with delivery at gestational age ≥ 22^+0^ weeks between 2014 and 2019 were included.

**Results:**

In the main cohort of expectantly managed triplet pregnancies (*n* = 106), 98% (312/318) of infants were liveborn with a mean gestational age at birth of 32^+3^ weeks and a mean birthweight of 1,726 g. Nine percent (*n* = 29) suffered from severe neonatal morbidity, and 4% (*n* = 12) died during the neonatal period. In the reduced cohort (*n* = 13 pregnancies), all infants were liveborn (*n* = 22). Mean gestational age at birth (36^+0^ weeks) and mean birthweight (2,444 g) were higher than in the expectantly managed cohort (*P* < 0.01 for both comparisons). There were no cases of severe neonatal morbidity (*P* = 0.24) or mortality (*P* = 1.00).

**Conclusion:**

Overall neonatal survival from 22^+0^ weeks of gestation in expectantly managed triplet pregnancies in Sweden was high. Nine out of 10 infants did not suffer from severe neonatal morbidity. Fetal reduction was performed in only a very small number of cases and was associated with higher gestational age at birth and higher birth weight.

## Introduction

During the last decades, multifetal birth rates have increased significantly in high-income countries, mainly due to assisted reproductive technologies (ART) (1–3). The Swedish regulation on in vitro fertilization (IVF) was revised in 2003, and single embryo transfer was set as normal routine to reduce the risk of multifetal pregnancies (4). The proportion of triplet deliveries in Sweden has been stable at about 0.2‰ for the past two decades (5). The risk of miscarriage as well as of fetal and neonatal death and neonatal and infant morbidity, mainly related to chorionicity and preterm birth, is increased in multifetal pregnancies (6). Maternal complications associated with multifetal pregnancy are, for example, gestational diabetes, hypertensive disorders of pregnancy, intrahepatic cholestasis, anemia, major obstetric hemorrhage, and cesarean section (7–10).

Fetal reduction is a procedure where the number of fetuses in a multifetal pregnancy is reduced by one or more (11). Fetal reduction has been shown to reduce the risk of preterm birth but carries an increased risk of miscarriage. A combined observational study and systemic review showed an increased risk of miscarriage after fetal reduction, but a decrease in very preterm birth (defined as gestational age [GA] <32^+0^ weeks) from 28 to 10% (12). Other retrospective studies have shown similar results, with significantly prolonged pregnancies after fetal reduction compared to expectantly managed triplet pregnancies (13, 14).

It is still uncertain whether multifetal pregnancy reduction carries a significant benefit in the context of contemporary maternity and neonatal care (15). Information regarding expected outcomes nationwide or for countries with similar maternal and infant care is important when counseling women pregnant with triplets regarding the possibility of reduction or expectant management.

The primary aim of this study was to describe maternal, pregnancy, and neonatal outcomes in expectantly managed triplet pregnancies using national data from Sweden. The secondary aim was to compare these outcomes in expectantly managed triplets with triplet pregnancies where fetal reduction had been performed.

## Materials and methods

In this nationwide cohort study of triplet pregnancies, triplet deliveries at GA 22^+0^ weeks or later from 1 January 2014 to 31 December 2019, registered in the Swedish Pregnancy Register, were included. The expectantly managed cohort included triplet pregnancies with a delivery of three infants, still-, or liveborn. The reduced cohort consisted of triplet pregnancies where fetal reduction had been performed exclusively in order to reduce the number of fetuses and not due to fetal complications. The reduced cohort was identified from the Fetal Therapy Register and linked to the Swedish Pregnancy Register. Data from both cohorts were linked to the Swedish Neonatal Quality Register (SNQ) to retrieve detailed information on neonatal outcomes (16–18).

Antenatal care in Sweden, including fetal therapy, is standardized and free of charge. High-risk pregnancies and deliveries are centralized to university hospitals. According to Swedish law, civil registration of all deliveries or pregnancy losses at GA 22^+0^ weeks or later is mandatory, while a pregnancy loss until GA 21^+6^ is defined as miscarriage (19). Swedish national guidelines from 2016 recommend active perinatal care for preterm births ≥ 23^+0^ weeks, whereas at 22^+0–6^ weeks, active care may be considered (20). All pregnant women are invited to an early second‐trimester ultrasound screening at 18–20 weeks of gestation, and approximately 97% of the pregnant population participate (21). In addition, during the years 2014–2019, an estimated up to one-half of the pregnant population underwent a first trimester screening ultrasound (16).

The Swedish Pregnancy Register is a national quality register, established in 2013 (16). It receives data from different sources. Most variables are obtained by direct transfer from the electronic medical records, which included approximately 91% of all births in Sweden (17 of 21 regions) during the study period.

The Fetal Therapy Register is a register at the Center for Fetal Medicine at Karolinska University Hospital, Stockholm. Manual registration in the Fetal Therapy Register is done for all women undergoing invasive fetal therapy in Sweden. Since 2013, highly specialized invasive fetal therapy has been centralized to one center in Sweden, Karolinska University Hospital. Reduction of multiple pregnancies in non-monochorionic pregnancies is formally not centralized to Karolinska University Hospital, but according to correspondence to all university hospitals in Sweden in 2020, multifetal reduction has probably not been performed outside Karolinska University Hospital in recent years.

SNQ is a national quality register, established in 2001 (17). It includes all infants born alive in Sweden who are admitted for neonatal care within 27 days after birth (around 11,500 annual admissions corresponding to 10% of all births in Sweden) and delivery room deaths. Completeness in SNQ for preterm infants in Sweden has been found to be excellent (17).

### Study population

**Figure 1:**
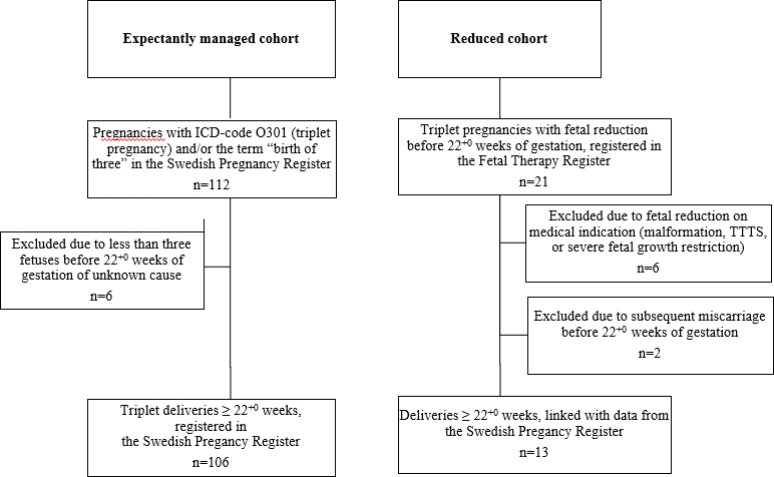
Flow diagram of the study protocol.

There were 112 triplet pregnancies with the ICD-10 (International Classification of Diseases) code O30.1 (triplet pregnancy) at any time during pregnancy and/or the term ‘birth of three’ in the Swedish Pregnancy Register (Figure 1). Six of those pregnancies were confirmed triplets by ultrasound early in pregnancy, but already at later ultrasound examinations before GA 22^+0^ weeks, less than three fetuses were registered. As we did not know the underlying cause for this, and with the intention to study only ongoing triplet pregnancies confirmed at GA ≥22^+0^ weeks, these six pregnancies were excluded. Thus, 106 triplet pregnancies formed the expectantly managed cohort.

For the reduced cohort, data on all triplet pregnancies where fetal reduction had been performed before GA 22^+0^ weeks during the study period were identified from the Fetal Therapy Register (*n* = 21). Six pregnancies, where fetal reduction had been performed due to fetal indications such as twin-to-twin transfusion syndrome or fetal malformation, and two pregnancies with miscarriage before GA 22^+0^ weeks after fetal reduction were excluded from the cohort (Figure 1). Thus, only ongoing pregnancies at GA ≥22^+0^ weeks after fetal reduction were included. The final reduced cohort consisted of 13 pregnancies.

The sample selection is illustrated as a flowchart in Figure 1.

Maternal outcomes obtained from standardized delivery records were the mode of delivery and estimated blood loss. Further, we analyzed blood transfusion, preeclampsia, and significant pregnancy complications presented as a composite variable including maternal blood loss in need of transfusion, placental abruption, preeclampsia, gestational hypertension, gestational diabetes, and liver disease during pregnancy. ICD-10 diagnostic codes and procedure codes used to identify these outcomes are presented in Supplementary Table 1.

**Table 1:** Maternal characteristics in the expectantly managed and the reduced cohort, respectively.

Maternal characteristics	Expectantly managed cohort *n* = 106 pregnancies	Reduced cohort *n* = 13 pregnancies	*P*
Age (years), mean ± SD	33 ± 6	34 ± 4	0.41^a^
*Data missing, n (%)*	*0*	*1 (7.7)*	
BMI, kg/m^2^, mean ± SD	26 ± 5	23 ± 4	0.046^a^
*Data missing, n (%)*	*0*	*3 (23.1)*	
Educational level, y, *n* (%)			
≤ 9	7 (6.6)	0 (0)	0.19^b^
10–12	32 (30.2)	2 (15.4)	
≥12	41 (38.7)	9 (69.2)	
*Data missing, n (%)*	*26 (24.5)*	*2 (15.4)*	
Country of birth, *n* (%)			
Sweden	53 (50.0)	7 (53.8)	0.97^b^
Non-Sweden	36 (34.0)	4 (30.8)	
*Data missing, n (%)*	*17 (16.0)*	*2 (15.4)*	
Nulliparous, *n* (%)	47 (44.3)	6 (46.1)	0.98^b^
*Data missing, n (%)*	*5 (4.7)*	*0*	
ART, *n* (%)	33 (31.1)^d^	8 (61.5)^e^	< 0.01^b^
*Data missing, n (%)*	*16 (15.1)*	*3 (23.1)*	
Smoking, early pregnancy, *n* (%)	4 (3.8)	0	1.0^c^
*Data missing, n (%)*	*19 (17.9)*	*2 (15.4)*	
Any significant pre-existing maternal disease, *n* (%)^f^	6 (5.7)	2 (15.4)	0.23^c^
*Data missing, n (%)*	*13 (12.3)*	*1 (7.7)*	

SD: standard deviation; BMI: body mass index; ART: assisted reproductive technology; IVF: in vitro fertilization.

a Calculated with Mann Whitney *U*-test.

b Calculated with Chi-square test.

c Calculated with Fisher’s exact test.

d Ovulation stimulation *n* = 13; IVF *n* = 20.

e Ovulation stimulation *n* = 4; IVF *n* = 4.

f Cardiovascular disease, pre-gestational diabetes, history of thrombosis, SLE, kidney disease or chronic hypertension; according to check-boxes filled out at first antenatal visit.

Pregnancy outcomes were stillbirth, GA at birth, birth weight, and small for gestational age (SGA; defined as a birthweight more than two SDs below the mean in a Swedish reference for normal fetal growth) [20]. Neonatal outcomes included neonatal mortality and the incidence of severe neonatal morbidity, analyzed as a composite variable which included intraventricular hemorrhage (IVH) grade 3 and 4, sepsis, necrotizing enterocolitis (NEC), bronchopulmonary dysplasia (BPD), and/or treated retinopathy of prematurity (ROP). Other outcomes were: Apgar at 5 min, neonatal resuscitation at birth, umbilical artery pH, and admission to the neonatal care unit. In SNQ, the definition of neonatal resuscitation included the need of mask ventilation, administration of continuous positive airway pressure (CPAP), intubation, cardiac compressions, correction of acidosis, or administration of epinephrine.

As a core outcome set for multiple pregnancies according to the CoRe Outcomes in WomeN’s health (CROWN) initiative has not been established yet, we chose important outcomes that have been reported in previous studies on multifetal pregnancies (22).

### Statistical analyses

Descriptive statistics were applied to present outcome variables in the cohorts. Outcomes were presented as numbers and percentages, mean ± standard deviation (SD), or median and interquartile range (IQR). Chi-square and Fisher’s exact test were applied to analyze differences in categorical data. Mann-Whitney *U*-test was carried out for the comparison of continuous, non-parametric data, between the two cohorts. The statistical software IBM SPSS statistics 25 was used for all statistical analyses. A *P*-value <0.05 was considered to be statistically significant.

### Ethical approval

Ethical permission for this study was given by the Swedish Ethical Review Authority with registration number: 2019-05160.

## Results

**Figure 2:**
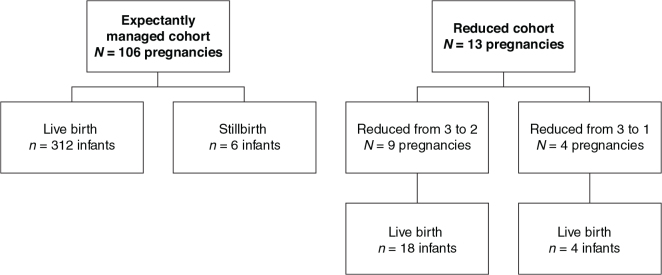
Number of pregnancies, fetuses and live- and stillbirths in expectantly managed and reduced triplet pregnancies.

In the expectantly managed cohort of 318 fetuses, six infants were stillborn (Figure 2). The reduced cohort consisted of 13 pregnancies with initially 39 fetuses before fetal reduction. Of those, the number of fetuses was reduced from three to two in nine pregnancies, and from three to one in four pregnancies, respectively. All 22 infants were liveborn (Figure 2).

Maternal age, parity, smoking in early pregnancy, level of education, country of birth, and pre-pregnancy comorbidity were not significantly different between women with expectantly managed and reduced triplet pregnancies (Table 1). In contrast, the utilization of ART was significantly less common in the expectantly managed (31% [33/106]) than in the reduced cohort (62% [8/13], *P* < 0.01). Moreover, maternal BMI in early pregnancy was slightly higher in expectantly managed (26 ± 5 kg/m^2^) compared to reduced triplet pregnancies (23 ± 4 kg/m^2^; *P* = 0.046).

Pregnancy and maternal outcomes are presented in Table 2. In the expectantly managed cohort, stillbirth of at least one infant occurred in three pregnancies. There was one pregnancy each with three, two and one stillborn fetus(es), respectively. Almost all women delivered by cesarean section (97% [103/106]). The median estimated blood loss was 700 mL (IQR 500–1090 mL), and 15% (16/106) of the women received a blood transfusion. Nine percent (9/106) of the women suffered from preeclampsia, and 33% (35/106) suffered from significant pregnancy complications. In contrast, in the reduced cohort, no infant was stillborn (*P* = 1.00), a lower proportion of women was delivered by cesarean section (31% [4/13], *P* < 0.01), and the median estimated blood loss was lower (440 mL [IQR 333–813 mL], *P* = 0.02). The proportion of pregnancy complications was lower than in the expectantly managed cohort, but without reaching statistical significance (7.7%, *P* = 0.11).There were no cases of maternal venous or pulmonary embolism and no cases of maternal death during pregnancy or the postnatal period in either group. Overall, consideration should be given to the very small number of pregnancies in the reduced cohort.

**Table 2:** Maternal and pregnancy outcomes in the expectantly managed and the reduced cohort, per pregnancy.

Outcome per pregnancy	Expectantly managed cohort *n* = 106 pregnancies	Reduced cohort *n* = 13 pregnancies	*P*
Pregnancies with stillbirth of at least one infant, *n* (%)	3 (2.8)	0	1.00^a^
Cesarean section, *n* (%)	103 (97.2)	4 (31)	< 0.01^a^
Total postpartum hemorrhage (mL), median (IQR)	700 (500–1,090)	440 (333–813)	0.02^b^
*Data missing, n*	*3 (2.8)*	*5 (38.5)*	
Blood transfusion to mother, *n* (%)	16 (15.1)	0	0.21^a^
Preeclampsia, *n* (%)	10 (9.4)	1 (7.7)	1.00^a^
Any significant pregnancy complication^c^, *n* (%)	35 (33.0)	1 (7.7)	0.11^a^

IQR: interquartile range.

a Calculated with Fisher’s exact test.

b Calculated with Mann-Whitney *U* test.

c Maternal blood loss in need of blood transfusion, placental abruption, preeclampsia, gestational hypertension, gestational diabetes, liver disorder in pregnancy.

Pregnancy and neonatal outcomes for liveborn infants (*n* = 312) are presented in Table 3. In the expectantly managed cohort, the mean GA at birth was 32^+3^ weeks (min – max: 22^+0^ – 36^+1^ weeks). Six percent (17/312) of the infants were born before 28^+0^ weeks of gestation. The mean birthweight was 1,726 ± 474 g, and 34% (106/312) were born SGA. Nine percent (29/312) had an Apgar score <7 at 5 min, and 92% (287/312) of the infants were admitted to neonatal care, where the median length of stay was 21 days (IQR 14–37 days). Sixty-three percent (196/312) of the infants needed resuscitation at birth, and 9% (29/312) suffered from severe neonatal morbidity. Four percent (12/312) of the infants died during the first 28 days of life, whereof 10 during the early neonatal period (0–7 days). Seven of the 12 infants who died had been born extremely preterm (22^+0^–26^+1^ weeks), two other infants had lethal malformations, and the remaining three had been born between 28 and 32 weeks of gestation (whereof two with extreme fetal growth restriction).

**Table 3:** Pregnancy and neonatal outcomes in the expectantly managed and the reduced cohort, per liveborn infant.

Outcome per liveborn	Expectantly managed cohort *n* = 312 liveborn infants	Reduced cohort *n* = 22 liveborn infants	*P*
Gestational age at birth			
Mean (± SD), days	227 ± 17.4	252 ± 17.8	<0.01^a^
Median (min – max), weeks ^+ days^	33 ^+ 1^ (22^+0^ – 36 ^+1^)	36 ^+6^ (32^+2^ – 40^+0^)	
Weeks, *n* (%)			
22^+0^ to 27^+6^	17 (5.4)	0	
28^+0^ to 31^+6^	73 (23.4)	0	
32^+0^ to 36^+6^	222 (71.2)	12 (54.5)	
≥37^+0^	0	10 (45.5)	
*Data missing, n (%)*	*0*	*0*	
Birth weight (grams)			
Mean ± SD	1,726 ± 474	2,444 ± 631	<0.01^a^
*Data missing, n (%)*	*2 (0.6)*	*1 (4.5)*	
Small for gestational age, *n* (%)	106 (34.0)	4 (18.2)	0.32^b^
*Data missing, n (%)*	*2 (0.6)*	*3 (13.6)*	
Boys, *n* (%)	168 (53.8)	14 (63.6)	0.37^c^
*Data missing, n (%)*	*0*	*2 (9.1)*	
Apgar score at 5 min, *n* (%)			
<4	9 (2.9)	0	0.06^c^
<7	20 (6.4)	0	
*Data missing, n (%)*	*11 (3.5)*	*4 (18.2)*	
Resuscitation at birth (mask or CPAP ventilation, intubation, heart massage, correction of acidosis, epinephrine), *n* (%)	196 (62.8)	4 (18.2)	<0.01^b^
Admission for neonatal care, *n* (%)	287 (92.0)	7 (31.8)	<0.01^c^
Length of stay at neonatal care unit in days (of those admitted), median (IQR)	21 (14–37)	14 (8–16)	<0.01^a^
Any severe neonatal morbidity (BPD, NEC, IVH 3 and 4, sepsis and/or treated ROP), *n* (%)	29 (9.3)	0	0.24^c^
Alive at 28 days of life, *n* (%)	300 (96)	22 (100)	1.00^b^

Small for gestational age: a birth-weight *z* score more than 2 SDs below the mean in a Swedish reference for normal fetal growth.

NA: not applicable; CPAP: continuous positive airway pressure; IQR: interquartile range; BPD: bronchopulmonary dysplasia; NEC: necrotizing enterocolitis; IVH: intraventricular hemorrhage; ROP: retinopathy of prematurity.

a Calculated with Mann-Whitney *U* test.

b Calculated with Fisher’s exact test.

c Calculated with Chi-square test.

In the reduced cohort – compared to the expectantly managed cohort – mean GA at birth was significantly higher (36^+0^ weeks [min – max: 32^+2^ – 40^+0^ weeks], *P* < 0.01) as was the mean birthweight (2,444 ± 631 g, *P* < 0.01), but there was no significant difference in the proportion of SGA births (18%; *P* = 0.32). Fewer infants were admitted to neonatal care (32%; *n* < 0.01), with a shorter median stay (14 days [IQR 8–16]; *P* < 0.01). Fewer infants needed resuscitation at birth (18%; *P* < 0.01), and none suffered from severe neonatal morbidity (*P* = 0.24). All infants were alive at 28 days of life (*P* = 1.0).

## Discussion

In a country with excellent access to high-quality maternity, delivery, and neonatal care, all infants in a nationwide cohort of expectantly managed triplet pregnancies were born preterm, whereof 6% extremely and 23% very preterm (22^+0^ – 27^+6^ weeks and 28^+0^ – 31^+6^ weeks of gestation, respectively). Perinatal survival rates were high, with 98% being liveborn, and 96% of liveborn infants being alive at 28 days of life. Nine out of 10 infants did not suffer from severe neonatal morbidity. Fetal reduction was performed in only a very small number of cases. In the reduced cohort, no infants were born extremely or very preterm, and mean birthweight and gestational age at birth were significantly higher compared to the expectantly managed cohort. There were no cases of severe neonatal morbidity or mortality and only one case of significant maternal pregnancy complication. However, consideration should be given to the very small number of pregnancies in the reduced cohort.

The proportion of pregnancies conceived by ART was high in the expectantly managed cohort (31%). This is in sharp contrast to the overall proportion of pregnancies conceived by ART in deliveries in Sweden, which was 3.8% during the years 2014–2019 (23). Despite the routine of single embryo transfer in the case of IVF in Sweden, multiple pregnancies still occur (24). Further, women can travel abroad for ART procedures with multiple embryo transfer. The proportion of pregnancies conceived by ART was significantly higher in the reduced cohort (62%), which is in line with previous studies, also showing that women who had a fetal reduction were substantially more likely to have used ART than were those who did not undergo fetal reduction (25, 26).

In the expectantly managed cohort, the proportion of cesarean sections was high (97%), which is in line with international guidelines, suggesting a planned cesarean section at 35 weeks of gestation in uncomplicated triplet pregnancies (27). A high proportion of women suffered from significant pregnancy complications (33%), which is comparable to other studies (7). In the reduced cohort, there was a significantly lower proportion of cesarean sections, and a non-significant lower proportion of maternal pregnancy complications probably due to the small sample size, which is expected and also in line with earlier studies (28). It might be considered controversial to test for a group difference concerning the proportion of cesarean sections, as it can be confounding by indication. However, to give birth vaginally or by cesarean is important from a clinical perspective, and thus, data are shown.

In contrast to our study, most other studies report deliveries from 24^+0^ rather than 22^+0^ weeks of gestation. Nevertheless, the rate of stillbirth and neonatal death in expectantly managed triplets in our study (2 and 4%, respectively) is comparable to or even slightly lower than in other studies. Curado et al. found an incidence of stillbirth of 2% in trichorionic triamniotic (TCTA) and 5% in dichorionic triamniotic (DCTA) triplets (due to mainly twin to twin transfusion syndrome, TTTS) (29). The rate of neonatal death has earlier been described to be twice that of stillbirth in both DCTA and TCTA triplets, which was also in line with our findings (29). While the rate of stillbirth is higher in triplets compared to singletons, several studies have found that neonatal morbidity and mortality in triplets were comparable to matched twins and singletons, highlighting the significance of prematurity and birthweight for these outcomes (15, 30, 31).

There were no significant differences in the proportion of severe neonatal morbidity or neonatal mortality between the expectantly managed and the reduced cohort. However, the non-significance is most probably due to the small number of neonates in the reduced cohort.

A main strength of the study is the nationwide design. The 106 triplet pregnancies in the expectantly managed cohort cover 84% of all triplet pregnancies ≥ 22^+0^ weeks of gestation when compared to data from Statistics Sweden during the years 2014–2019 (5). The coverage rate for transfer of data from the electronic medical records to the Swedish Pregnancy Register during the study period was 91%, which may explain the main part of the difference.

To be mentioned as a second strength, also data on fetal reduction from the Fetal Therapy Register can be considered as nationwide. Thus, this study provides combined data from three registers with prospectively registered information. The coverage rates of the registers are exceptionally high, with high quality of data.

Firstly, the significant limitation in our study is the lack of information on chorionicity. Secondly, it was not possible to follow triplet pregnancies including miscarriages from early gestation through registers in Sweden, as a first trimester ultrasound is not offered to all pregnant women. In contrast, it was possible to identify basically all triplet pregnancies with a delivery ≥ 22^+0^ weeks of gestation in the country. Thirdly, the number of pregnancies in the reduced cohort was small. Triplet pregnancies are rare events, and fetal reductions with the only indication to reduce the number of fetuses are even more rare, especially in a country where single embryo transfer is routine in the case of IVF. However, the study population represents almost the whole Swedish population of expectantly managed and reduced triplets, delivered ≥ 22^+0^ weeks of gestation. Thus, that fetal reduction with the only indication to reduce the number of fetuses was a rare event in our study (and thus in Sweden) is an important finding itself. Fourthly, data on some outcomes were missing to a higher extent in the reduced compared to the expectantly managed cohort, as for example Apgar score at 5 min of age (missing in 4/22 [18.2%] and 11/312 [3.5%] of infants, respectively), making comparisons between the cohorts more difficult.

According to Statistics Sweden, the proportion of triplet deliveries ≥ 22^+0^ weeks of gestation was 0.018% during the years 2014–2019 in Sweden (5). The proportion of triplet pregnancies in our study was 0.017%. In contrast, although declining from the 1998 peak with a proportion of 0.19% in the US, the proportion of triplet deliveries in the US was still almost fivefold that in Sweden in 2019 (0.084%) (32). Preventing triplet pregnancies by a routine of single embryo transfer in case of IVF or Intracytoplasmic sperm injection (ICSI) and by careful monitoring in case of ovarian stimulation is the method of choice to reduce maternal and infant morbidity and mortality. However, triplet pregnancies will always occur to some proportion, and knowledge about management and outcomes is important.

With the limitation that we do not have any data about miscarriage rates before GA 22^+0^ weeks in expectantly managed triplets in our cohort, our data regarding triplets born GA ≥ 22^+0^ weeks are in line with previous studies (15, 26, 28, 33). When the top priority is liveborn infants, expectant management seems to be the best choice. When the priority is to minimize prematurity – and probably also neonatal and long-term morbidity – the most advisable option seems to be fetal reduction. Women (and partners) with a triplet pregnancy ultimately must make decisions with potentially lifelong consequences, and careful counseling is of utmost importance (28).

## Conclusion

While overall neonatal survival from 22^+0^ weeks of gestation in expectantly managed triplet pregnancies in Sweden was high, all infants were born preterm. Every tenth infant and every third mother suffered from severe neonatal morbidity or pregnancy complications, respectively. Fetal reduction was performed in only a very small number of cases and was associated with higher gestational age at birth and higher birth weight. In an international context, rates of multifetal births are very low in Sweden.
