# Supplementary material for: Maternal, pregnancy and neonatal outcomes in triplet pregnancies in Sweden – a nationwide cohort study
Source: Ups J Med Sci. 2023 Jul 17;128:10.48101/ujms.v128.9473. doi: 10.48101/ujms.v128.9473 (PMC10395261; doi:10.48101/ujms.v128.9473)
Supplement: Supplementary file 1 [file UJMS-128-9473-s001.pdf]

SUPPLEMENTARY MATERIAL

Table 4. ICD 10-codes for maternal outcomes

Condition	ICD 10-code
Blood transfusion to mother	DR029 Transfusion of erythrocytes
Placental abruption	O45 Premature separation of placenta [abruptio placentae]
Preeclampsia	O14 Pre-eclampsia<br>O15 Eclampsia
Hypertension	O13.9 Gestational [pregnancy-induced] hypertension
Gestational diabetes	O24.4 Diabetes mellitus in pregnancy
DVT/PE	O22.3 Deep phlebothrombosis in pregnancy<br>I26 Pulmonary embolism
Intrahepatic cholestasis	O26.6 Liver disorders in pregnancy, childbirth and the puerperium
